# Validation of the Norwegian versions of the Implementation Leadership Scale (ILS) and Multifactor Leadership Questionnaire (MLQ) in a mental health care setting

**DOI:** 10.1186/s40359-022-00725-8

**Published:** 2022-02-08

**Authors:** Nora Braathu, Erlend Høen Laukvik, Karina M. Egeland, Ane-Marthe Solheim Skar

**Affiliations:** Norwegian Centre for Violence and Traumatic Stress Studies (NKVTS), Gullhaugveien 1-3, 0484 Oslo, Norway

**Keywords:** Evidence-based practice, Mental health, Implementation climate, Implementation strategies, PTSD, Transformational leadership, Implementation leadership

## Abstract

**Background:**

The implementation of evidence-based practices (EBPs) is of crucial importance in health care institutions and requires effective management from leaders. However, there is a lack of assessment tools sufficient to evaluate the degree to which the employees´ rate how well their leaders are at implementing EBPs. This emphasises the need for validated and widely used scales relevant for EBPs.

**Methods:**

The current study evaluated the psychometric properties of the Multifactor Leadership Questionnaire (MLQ) and Implementation Leadership Scale (ILS) in a Norwegian mental health care setting.

**Results:**

Results from confirmatory factor analyses indicate that the MLQ and ILS are valid instruments for measuring general and implementation leadership. The scales demonstrate good convergent validity. In addition, attitudes towards EBPs did not seem to be associated with the ILS and MLQ, further supporting the applicability of the instruments.

**Conclusions:**

The two scales demonstrate good psychometric properties in a Norwegian mental health care setting, which suggests that the MLQ and ILS are valid and reliable tools for measuring leadership in an implementation setting. More research is greatly needed to disentangle the link between perceived leadership and objective measures of successful implementation of EBPs.

*Trial registration* NSD 690,133, NSD 60,059/3/OOS.

## Background

Effective leadership has been identified as an important factor associated with the successful implementation of evidence-based practices (EBPs) in mental health services [1–3]. The focus on implementing EBPs in the healthcare system started in the 1990’s [4]. Over the years, it has subsequently been shown that successfully implementing EBPs may lead to better and more effective health care services, with lower costs, higher job satisfaction among clinicians, and higher patient satisfaction [5–7]. Leaders are an important part of any implementation process, as they can influence the organisational climate at the workplace, cooperation between team members, and employee’s attitudes towards the EBP [8–11]. With the growing interest in the role that leadership plays in effective EBP implementation, there is a need for establishing reliable and valid measures to assess leadership behaviours that relate to successful implementation.

In the quest to discern the leader’s role in the implementation of EBPs, there was initially a focus on identifying general leadership behaviours associated with different implementation outcomes [11, 12], using leadership concepts such as the Full-Range Leadership (FRL; [13]) theory. FRL is one of the most widely used leadership theories. It describes different leadership behaviours such as transformational leadership, in which leaders motivate and encourage employees; transactional leadership, where the leader rewards and punishes employees based on performance; and non-leadership, where the leader has a more “hands off” approach and avoids making decisions [14, 15]. Several studies have shown a positive relation between transformational leadership and different implementation outcomes, such as employees’ attitudes towards EBPs [16], motivation [17], turnover intention [18], burnout [19], and overall improved performance at all levels of the workplace [20–22].

The Multifactor Leadership Questionnaire (MLQ) is based on the FLR leadership theory and is likely the most frequently used scale to measure leadership [23]. The scale has been psychometrically validated several times, showing acceptable scores (ranging from α = 0.78 to 0.94; [21–24]). The MLQ has also previously been validated in a Norwegian sample [24], where each subscale showed adequate psychometric properties (α = 0.62—0.84). However, the validations differ as a result of researchers altering the original factor structure by either combing or excluding certain factors or items [25, 27]. The original structure consists of nine subscales, where *idealised influence* was separated into *behaviours* and *attributed charisma* [31]. However, several researchers treat *idealised influence* as one factor [23, 25, 27], and the scale has accordingly been broadly used as an eight-factor scale, consisting of *idealised influence*, *inspirational motivation*, *intellectual stimulation*, *individualised consideration*, *contingent reward*, *management-by-exception (active)*, *management-by-exception (passive)* and *laissez-faire*. However, although most use the eight- or nine-factor models [25, 28, 29], some have rearranged the subscales into categories different from what was originally proposed (i.e., two subscales measuring non-leadership; [26, 30]). These alterations could cause misunderstandings when using the MLQ in relation to implementation outcomes. The creators of the scale have recommended individually analysing each of the eight subscales of the MLQ, with the exception of the transformational leadership subscales, which can be combined [31]. The current study therefore considers the MLQ as consisting of eight subscales: four subscales measuring transformational leadership, three subscales measuring transactional leadership and one measuring non-leadership.

While interest in the relationship between these more general leadership concepts and successful EBP implementation was increasing, research on strategic leadership behaviours related to implementation was lacking. Building on the growing evidence base that general leadership behaviours relate to effective EBP implementation, scientists have turned their interest towards specific leadership behaviours that may be more proximally related to successful EBP implementation. Newer research has established that such specific, or strategic, as often termed, leadership behaviours may provide additional explanatory value in the investigation into how leadership relates to key implementation outcomes [8, 32, 33].

The focus on identifying strategic leadership behaviours for implementation coincides with a more general call within the implementation research field to develop simple, brief and psychometrically sound implementation measures [34, 35]. In 2014, Aarons, Ehrhart and Farahnak developed the *Implementation Leadership Scale* (ILS), drawing from a broad base of theory and research on implementation, leadership, and organisational climate [11]. Results from studies investigating the effect of strategic leadership behaviours (i.e., implementation leadership) have revealed that these promote organisational change [12]. This is consistent with findings which report that employee-ratings on the ILS correlate with factors considered to be important during the implementation of EBPs and their sustainment [36].

The ILS was initially developed in the U.S., and has been validated several times [37–39]. In investigations using both employee-ratings and leader self-ratings, the ILS has shown excellent psychometric properties in multiple sectors [11, 39–42]. The ILS contains four subscales, including *proactive leadership*, *knowledgeable leadership*, *supportive leadership* and *perseverant leadership*, and the suggested four-factor structure has been confirmed in all studies [5, 11, 37, 39]. Analysis of reliability has found internal consistency to be excellent (Cronbach’s α ranging from 0.92—0.98) [11, 39, 41]. Convergent validity has been investigated by correlating the ILS with the MLQ, finding moderate to high correlations (Pearson’s correlation ranging from 0.63 to 0.75) between the two leadership concepts [11]. Discriminant validity has been established by correlating the ILS to theoretically unrelated implementation concepts, such as the Evidence-based Practice Attitude Scale (EBPAS), finding zero to low correlations (Pearson’s correlations ranging from 0.05 to 0.4) [11]. Only two studies have investigated the psychometric properties of the ILS outside the U.S., doing so in China and Greece [5, 43]. Employee ratings have previously been used when investigating the psychometric properties in a U.S. context [11], and a similar investigation in a Norwegian context would provide further evidence for the relevance of the concept of implementation leadership.

Based on the abovementioned, it is clear that successful implementation of EBPs relies on effective leadership. To accurately and reliably measure elements of effective leadership important for EBP implementation, we need valid measures. The purpose of this study is to examine the psychometric properties of the Norwegian version of the MLQ and ILS. First, the factor structure and internal consistencies of the two scales will be explored. Secondly, we will examine the convergent and divergent validity of the MLQ and ILS. Based on previous findings regarding the ILS, we expect to find support for a four-factor model and high internal consistency for the total scale and all subscales. In addition, we anticipate that the ILS will have moderate to high correlations with the MLQ and subsequently low correlations with the EBPAS. In accordance with other studies, we expect to find support for an eight-factor model [44, 45], as well as similar results regarding convergent and divergent validity as hypothesised above.

## Method

### Procedure

The study took place as part of a national implementation of evidence-based treatment for post-traumatic stress disorder (PTSD) in Norwegian specialised mental health care clinics for adults (N = 25) and youth (N = 22) [46]. The data was collected in the context of the utilisation of the Leadership and Organisational Change for Implementation (LOCI) as an implementation strategy [46, 47]. Local health trusts were contacted via e-mail with an invitation to participate in the implementation project and research study. Participating clinics were included in the hybrid type II project [48] based on motivation and availability. Data was collected from leaders and therapists working at the local health trusts between 2018 and 2020. Participation was voluntary and informed consent was attained from all participants in the study.

### Participants

Participants were 804 therapists working at child or adult mental health clinics in Norway. The final sample size was 795 after removing missing data. Close to half (46.2%) of the participants were psychologists; 75 percent were females, and the average age was 43.9 years. Participants rated their leaders (N = 47) by filling out questionnaires regarding leadership, work climate and other measures relevant for the intervention. The average age for leaders was 49.7, and 55 percent had a background in psychology (Table 1).

**Table 1:** Participant demographics

	Therapists (N = 795)	Leaders (N = 47)
**Gender**		
Female	599 (75.3%)	29 (61.7%)
Male	169 (22.5%)	18 (38.3%)
**Age**		
Mean (SD)	43.8 (11.1)	49.7 (7.64)
Missing	114 (14.3%)	0 (0%)
**Education**		
Psychology	367 (46.2%)	26 (55.3%)
Social work	60 (7.5%)	8 (17.0%)
Nurse	53 (6.7%)	8 (17.0%)
Medicine	148 (18.6%)	5 (10.6%)
Other	89 (11.2%)	0 (0%)
Missing	78 (9.8%)	0 (0%)
**Years in current position**		
Mean (SD)	11.8 (9.56)	21.5 (18.3)
Missing	192 (24.2%)	0 (0%)

## Measures

### Implementation Leadership Scale (ILS)

ILS is a 12-item measure addressing leadership support for the usage of EBP [11]. It covers four different implementation leadership dimensions: *Proactive Leadership* describes the degree to which the leader anticipates and addresses implementation challenges; *Knowledgeable Leadership* refers to the degree to which a leader has a deep understanding of EBP and implementation issues; *Supportive Leadership* measures the degree of the leader’s support of followers’ adoption and use of EBP; and *Perseverant Leadership* refers to the degree to which the leader is consistent, unwavering, and responsive to EBP implementation. It is scored from 0 (not at all) to 4 (to a very great extent). The total ILS score is created by computing the mean of the four subscales. ILS was translated to Norwegian by an independent research group at the Regional Center for Children and Adolescent Mental Health (RBUP). The third and fourth author completed an additional back-translation. Both the initial translation and back-translation were done in close collaboration with the developers of the scale. There were only small differences between the two translations, and minor adjustments were made to align the translations.

### Multifactor Leadership Questionnaire (MLQ)

The MLQ is a 36-item questionnaire, measuring transformational and transactional leadership, as well as non-leadership [49]. Transformational leadership consists of four subscales. *Inspirational Motivation* measures how positive and motivated the leader is about the future, which may influence the employees’ feelings of motivation. *Idealised Influence* focuses on the leaders’ attributes, like perceived power, values and ideals, and underlines a collective sense of these mission and values [25]. *Intellectual Stimulation* refers to whether the leader introduces new methods of viewing issues and seeks different perspectives. Lastly, *Individualised Consideration* measures how well the leader considers individual needs and helps the employees develop their strengths.

Transactional leadership consists of three subscales. *Contingent Reward* is a leadership behaviour that focuses on clear, defined requirements, and rewards desired outcomes through economical or emotional advantages. In both *Management-by-exception active and passive*, the leader provides corrective action when they notice behaviours that deviate from the norm. In the active subscale, the leader actively monitors actions and intervenes before the deviations start occurring, while in the passive, the leader waits until the deviations have occurred [50]. Lastly, non-leadership is measured by *Laissez-faire*, which assesses the absence of leadership. The MLQ is scored from 0 (Not at all) to 4 (Frequently, if not always). A Norwegian translation was used in the current study [24].

### Evidence-based Practice Attitude Scale (EBPAS)

The EBPAS is a 15-item scale measuring mental health providers’ attitudes toward the adoption of evidence-based practices [51]. The scale has four subscales: *Appeal*, which describes the intuitive appeal of the practice; *Openness*, referring to openness to new practices; *Requirement*, the likelihood of adopting the EBP if required to do so; and *Divergence*, which refers to the new practice’s perceived divergence from usual practice. The EBPAS is scored on a 5-point Likert scale ranging from 0 (Not at all) to 4 (To a very great extent). The overall score is calculated by reversing the scores on the divergent subscale, and then averaging the items from all scales. A Norwegian translation was used in the current study [52]. The scale has shown adequate psychometric properties in a Norwegian sample of therapists working in mental health care services (α = 0.86; (56), α = 0.81; [53], and similar properties were found in the current study (15-items; α = 0.87, CI (95% bootstrapping based on 1000 samples) = 0.853– 0.884).

## Statistical analyses

### Internal consistency

Internal consistency was assessed by examining Cronbach’s alpha for all subscales and the total scale (Table 2).

**Table 2:** Summary statistics for the ILS total scale, subscales, and scale items and the MLQ subscales and scale items

ILS and MLQ	Mean	SD	*a*
***Implementation leadership subscales***			
**Proactive leadership**	2.05	1.01	.93
1. Developed a plan to facilitate EBP implementation	2.14	1.09	
2. Removed obstacles to implementation of EBP	2.02	1.07	
3. Established clear standards for implementation of EBP	1.99	1.09	
**Knowledgeable leadership**	2.21	1.10	.97
4. Is knowledgeable about EBP	2.20	1.13	
5. Able to answer staff questions about EBP	2.16	1.16	
6. Knows what he/she is taking about when it comes to EBP	2.26	1.13	
**Supportive leadership**	2.81	0.96	.96
7. Supports employee efforts to learn more about EBP	2.74	1.01	
8. Recognises and appreciates employee efforts	2.85	1.00	
9. Supports employee efforts to use EBP	2.83	1.00	
**Perseverant leadership**	2.49	0.96	.95
10. Perseveres through the ups and downs of implementing	2.51	1.00	
11. Carries on through the challenges of implementing EBP	2.56	0.98	
12. Reacts to critical issues regarding implementation of EBP	2.41	1.05	
**ILS total (12 items)**	2.39	0.88	.96
***Multifactor leadership questionnaire***			
**Individualised consideration**	2.62	0.87	.86
1. Spends time teaching and coaching	2.19	1.05	
2. Treats you as an individual rather than just a member of the group	3.12	0.97	
3. Considers that you have different needs, abilities, and aspirations from others	2.73	1.03	
4. Helps you develop your strengths	2.45	1.09	
**Intellectual stimulation**	2.60	0.87	.91
5. Re-examines critical assumptions to question whether they are appropriate	2.49	1.03	
6. Seeks differing perspectives when solving problems	2.83	0.94	
7. Gets you to look at problems from many different angles	2.51	0.99	
8. Suggests new ways of looking at how to complete assignments	2.58	0.95	
**Inspirational motivation**	2.76	0.79	.84
9. Talks optimistically about the future	2.99	0.94	
10. Talks enthusiastically about what needs to be accomplished	2.85	1.00	
11. Articulates a compelling vision of the future	2.29	0.98	
12. Expresses confidence that goals will be achieved	2.89	0.88	
**Idealised influence**	2.72	0.81	.92
13. Instils pride in you for being associated with him/her	2.43	1.13	
14. Goes beyond self-interest for the good of the group	2.88	0.95	
15. Acts in ways that builds your respect	2.99	1.00	
16. Displays a sense of power and confidence	2.90	1.03	
17. Talks about his/her most important values and beliefs	2.41	1.02	
18. Specifies the importance of having a strong sense of purpose	2.53	0.99	
19. Considers the moral and ethical consequences of decisions	2.78	1.00	
20 Emphasises the importance of having a collective sense of mission	2.82	0.92	
**Transformational leadership total**	2.69	0.75	.96
**Contingent reward**	2.45	0.87	.86
21. Provides assistance in exchange for your efforts	2.80	1.06	
22. Discusses in specific terms who is responsible for achieving performance targets	2.19	1.05	
23. Makes it clear what you can expect to receive when performance goals are achieved	1.97	1.09	
24. Expresses satisfaction when you meet expectations	2.81	0.95	
**Management by exception active**	1.73	0.96	.89
25. Focuses attention on irregularities, mistakes, exceptions, and deviations	1.89	1.01	
26. Concentrates his/her full attention on dealing with mistakes, complaints, and failures	1.53	1.18	
27. Keeps track of all mistakes	1.67	1.15	
28. Directs your attention to failures to meet standards	1.85	1.07	
**Management by exception passive**	0.82	0.82	.85
29. Fails to interfere until problems become serious	0.90	1.02	
30. Waits for things to go wrong before taking action	0.68	0.93	
31. Shows that he/she is a firm believer in “If it ain’t broke don’t fix it”	1.08	1.06	
32. Demonstrates that problems must become chronic before taking action	0.61	0.92	
**Laissez-faire**	0.66	0.78	.88
33. Avoids getting involved when importance issues arise	0.50	0.83	
34. Is absent when needed	0.84	0.98	
35. Avoids making decisions	0.68	0.92	
36. Delays responding to urgent questions	0.64	0.91	

### Confirmatory factor analysis

Confirmatory factor analysis was conducted using MPlus 8.3 [54]. Fit indices (standardised root mean square residual = SRMR, root mean-square error of approximation = RMSEA, comparative fit index = CFI and Tucker-Lewis index = TLI) were used as indicators of validity. We used recommended cut-offs that indicate a good fit for validation: CFI & TLI ≥ 0.90, SRMR < 0.08, RMSEA < 0.08 [55, 56]. The CFA was conducted using the weighted least square mean and variance estimation (WLSMV), which is ideal for categorical data. As clinicians working in the same clinic had the same leader, we controlled for the multilevel, nested data structure.

### Convergent and discriminant validity

Discriminant and convergent validity were subsequently calculated by correlation analyses. To assess convergent validity, the MLQ and ILS were compared to each other. Divergent validity was measured by viewing the correlations between both aforementioned scales to the EBPAS subscale scores. This was done by using employee-ratings (clinicians working with patients) in specialised Norwegian mental health care services. The analyses were conducted using IBM SPSS Statistics for Windows, V.26 [57] and Rstudio [58].

## Results

### Reliability

The internal consistency of the ILS total scale (α = 0.96) and the four factors were excellent (ranging from α = 0.93–0.97; see Table 2). The internal consistency of Transformational Leadership was also excellent (α = 0.96). Individualised Consideration (*a* = 0.86), Intellectual Stimulation (*a* = 0.91), Inspirational Motivation (*a* = 0.84), Idealised Influence (*a* = 0.92), Contingent Reward (*a* = 0.86), Management-by-Exception Active (*a* = 0.89), Management-by-Exception Passive (*a* = 0.85), and Laissez-faire (*a* = 0.88) showed acceptable internal consistency.

## Confirmatory factor analysis

### Implementation leadership scale

**Fig. 1:**
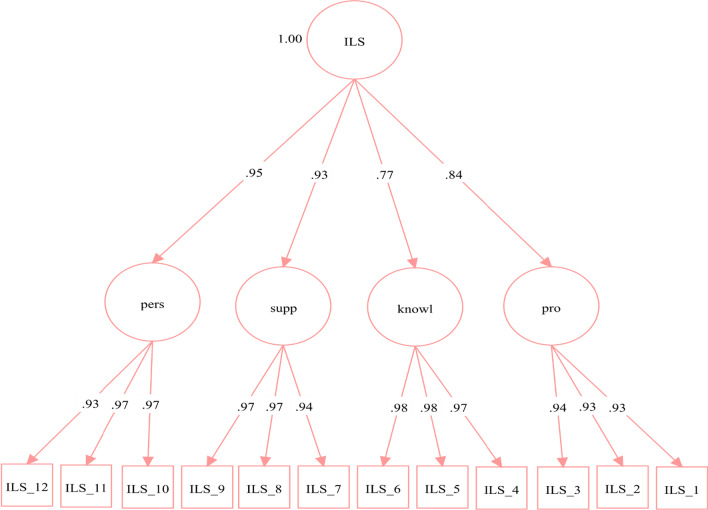
Standardised factor loadings for the ILS. The parameters are presented as standardised path coefficients. The circular shapes represent the factors, or subscales. The square boxes below represent the items. Arrows pointing from the factors to the items are the first-order factor loadings. Arrows from the latent construct (ILS) to the subscales are the second-order factor loadings. Pers = Perseverant, Supp = Supportive, Knowl = Knowledgeable, Pro = Proactive

As each subscale of the ILS is considered an indicator of overall implementation leadership, a higher order model was considered. The higher order CFA model demonstrated excellent fit (χ^2^ (48) = 112.575, *p* < 0.001; CFI = 0.999, TLI = 0.999; RMSEA = 0.043; SRMR = 0.010). Standardised factor loadings are displayed in Fig. 1, and all factor loadings were significant (*p*’s < 0.001). Nested data was controlled for by using the function cluster = and type = complex in Mplus and grouped by leaders (see Fig. 1).

### Multifactor Leadership Questionnaire

**Fig. 2:**
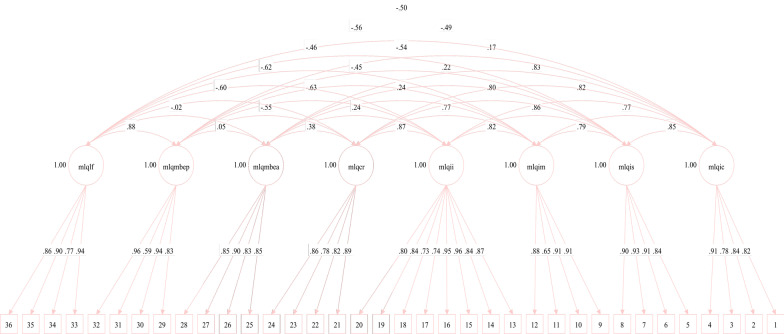
Standardised factor loadings for the MLQ—The parameters are presented as standardised path coefficients. The circular shapes represent the factors, or subscales. The square boxes below represent the items. Arrows pointing from the factors to the items are the factor loadings. mlqlf = Laissez-faire, mlqmbep = Management-by-exception passive, mlqmbea = Management-by-exception active, mlqcr = Contingent Reward, mlqii = Idealised Influence, mlqim = Inspirational Motivation, mlqis = Intellectual Stimulation, mlqic = Individualised Consideration

The eight-factor model also showed excellent fit (χ^2^ (566) = 1891.317, *p* < 0.001; CFI = 0.968, TLI = 0.964; RMSEA = 0.056; SRMR = 0.050). Nested data was controlled for in the same way as aforementioned. (See Fig. 2). As the previous Norwegian version of the MLQ had been validated as a three-factor structure [24], this was also done in the current study. Results revealed an unacceptable model fit (χ^2^ (591) = 16,024.869, *p* < 0.001; CFI = 0.559, TLI = 0.530; RMSEA = 0182; SRMR = 0.182) according to the recommended cut-off indices. This suggests that the 8-factor version validated in the current study is more suitable.

### Convergent and discriminant validity

**Fig. 3:**
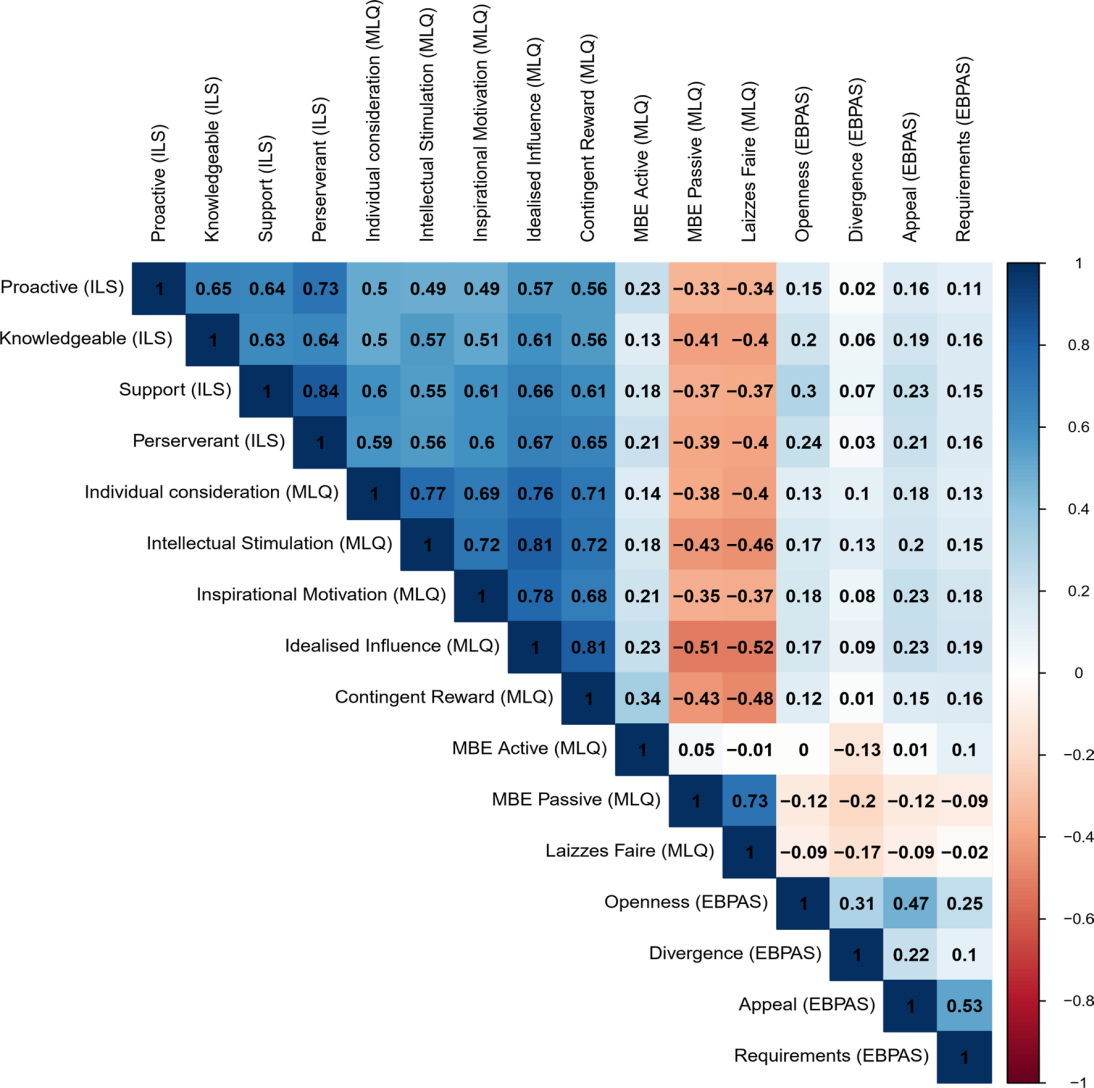
Correlation matrix including the ILS, MLQ and EBPAS. White squares indicate *p* > .05**.** The bar on the far right indicates the correlation between the subscales. Blue colors indicate a positive correlation, while orange colors indicate a negative correlation

The ILS subscales had moderate to high correlations with the score of the MLQ Transformational Leadership subscale and the Contingent Reward subscale, which is consistent with previous findings [11]. Correlations ranged from 0.49 to 0.57, as shown in Fig. 3. As predicted, the ILS and MLQ both had low correlations with the EBPAS subscales, with correlations ranging from -0.17 to 0.23 (see Fig. 3).

## Discussion

The aim of the current study was to investigate the psychometric properties of the Multifactor Leadership Questionnaire and Implementation Leadership Scale in a Norwegian clinical mental health care setting. Both general leadership [11] and implementation leadership [11] have been shown to be important for facilitating successful implementation. Results from the confirmatory factor analyses showed an excellent fit, supporting an eight-factor model for the MLQ and a four-factor model for the ILS. Regarding the ILS, the internal consistency was excellent for the total scale and for subscales. Results from the correlations indicate strong support for both convergent validity in regard to MLQ transformational leadership and discriminant validity in regard to EBPAS. Consistent with previous studies [11], the moderate to high correlations with the MLQ transformational leadership indicate that similar constructs are measured in both of the two scales, but not so similar that they would be considered identical. This suggests that the two scales can be used together to get an overall picture of behaviours important for effective implementation of EBPs. This is in line with theories suggesting that general and strategic leadership are different concepts, both influencing the implementation of EBPs [11]. Furthermore, the low correlations between the EBPAS and the ILS, and the EBPAS and the MLQ, support the hypothesis that they theoretically measure different constructs, which is in line with previous studies [11].

Research on the MLQ has varied in that researchers occasionally combine different subscales into one factor [27, 59], instead of assessing the subscales individually as done in the current study and according to recommendations from the developers of the scale [31]. Due to the acceptable values regarding internal consistency and expected results regarding convergent and discriminant validity, the findings indicate that the original eight-factor structure can be maintained. Furthermore, results indicate that each of the subscales constituting the scale measure different constructs within general leadership.

With measures such as the ILS and MLQ, we are able to assertively measure leadership behaviours assumed necessary to successfully implement changes that consequently improve patients’ well-being. Moreover, the validation of the MLQ is novel in regard to the factor structure. The previous validation in a Norwegian sample found a three-factor structure consisting of several subscales in each factor [24], while we found support for each subscale as individual factors. Previous research has suggested that the inconsistent findings regarding the MLQ subscales may occur due to heterogeneous samples of leaders from different cultural and professional backgrounds [28, 31]. The current sample is a quite homogenous group, consisting of Norwegian mental health service practitioners, mostly women aged 44. This supports previous findings regarding the individual factors of the MLQ [28]. Furthermore, findings from the current study add to the literature regarding the leadership and implementation of EBPs by including results from a Nordic sample. Overall, investigations into the psychometric properties of scales frequently used for measuring key implementation concepts, such as in this study, lays the foundation for gaining valid knowledge on the complex process of successfully implementing EBPs, which subsequently have large clinical implications as these factors influence patient outcomes [19].

The current study had a large sample size, spread across 43 clinics throughout the country. This variety is a clear strength, as it increases generalisability. A limitation of the current study was that data was only collected from participants in mental health clinics. We encourage future studies to investigate the psychometric properties of these scales in other sectors, and between different professions within these sectors. In addition, it would be interesting to compare results from different time points to examine the test–retest reliability. This could not be done in the current study as we expect the LOCI intervention to alter scores on the ILS and MLQ. Although results suggest that high scores on the transformational leadership and contingent reward subscales are positively correlated to factors of implementation leadership, it has not yet been established whether these leaders actually have been successful at implementing EBPs.

## Conclusion

As research has established leadership as an important factor for successful implementation, there is a need for efficient measures that assess both general and strategic leadership behaviours. The current study demonstrates that the Norwegian versions of the *Multifactor Leadership Questionnaire* and *Implementation Leadership Scale* are valid and reliable instruments for measuring general leadership and leadership in the context of EBP implementation, respectively. Results show that the subscales of transformational leadership and contingent reward correlate with all subscales of the ILS. Divergent validity analysis shows that the EBPAS is a theoretically different construct compared to the MLQ and ILS. More research is needed to further understand how different leadership behaviours, both general and strategic, relate to the successful implementation of EBPs.

## Data Availability

The dataset used in the current study is available from the corresponding author upon reasonable request.

## Abbreviations

EBP: Evidence-based practice
ILS: Implementation Leadership Scale
MLQ: Multifactor Leadership Questionnaire
EBPAS: Evidence-based Practice Attitudes Scale
PTSD: Post-traumatic stress disorder
